# Comparison of Bicuspid and Tricuspid Handmade Polytetrafluoroethylene Valved Conduits: Early and Mid-Term Results

**DOI:** 10.3390/jcm14061957

**Published:** 2025-03-13

**Authors:** Murat Çiçek, Fatih Özdemir, Okan Yurdakök, Oktay Korun, Mehmet Akif Önalan, Emine Hekim Yılmaz, Türkan Kudsioğlu, Numan Ali Aydemir

**Affiliations:** 1 Department of Pediatric Cardiovascular Surgery, Dr. Siyami Ersek Thoracic and Cardiovascular Surgery Training and Research Hospital, Istanbul 34668, Turkey; fatih.ozdemir.83@gmail.com (F.Ö.); okanyurdakok@gmail.com (O.Y.); mehmetakifonalan@gmail.com (M.A.Ö.); numanaydemir@gmail.com (N.A.A.); 2 Department of Cardiovascular Surgery, Faculty of Medicine Istanbul, Istanbul University-Cerrahpaşa, Istanbul 34320, Turkey; oktay_korun@hotmail.com; 3 Department of Pediatric Cardiology, Dr. Siyami Ersek Thoracic and Cardiovascular Surgery Training and Research Hospital, Istanbul 34668, Turkey; drehekim@gmail.com; 4 Department of Anesthesiology and Reanimation, Dr. Siyami Ersek Thoracic and Cardiovascular Surgery Training and Research Hospital, Istanbul 34668, Turkey; turkancoruh@gmail.com

**Keywords:** polytetrafluoroethylene, right ventricle outflow tract, handmade valved conduit

## Abstract

**Background:** In this study, we present our early and mid-term results using two different types of handmade polytetrafluoroethylene (PTFE) valved conduits in patients who require right ventricular outflow reconstruction. **Methods:** Between March 2021 and May 2024, 72 patients (30 males and 42 females; median age: 69 (IQR: 26–123) months) who underwent implantation of a handmade bicuspid or tricuspid valve PTFE conduit for right ventricular outflow reconstruction were retrospectively analyzed. Preoperative, postoperative, and follow-up echocardiograms were also evaluated. **Results:** The first postoperative echocardiography revealed that 11 (36.7%) patients had mild regurgitation, and 3 (10%) patients had moderate regurgitation in the bicuspid group initially, while only 7 (16.7%) of the patients in the tricuspid group had mild regurgitation (*p* = 0.004). None of the patients required reintervention in the early postoperative period because of conduit dysfunction. In the mid-term follow-up, the mean follow-up duration was 22.4 ± 11 months. PTFE-valved conduit dysfunction was observed in three patients in the bicuspid group, while no dysfunction was observed in the tricuspid group (*p* = 0.049). Even if the median peak gradient was found to be slightly higher in the tricuspid group [15 (IQR: 0–25) vs. 0 (IQR: 0–15)] (*p* = 0.032), no conduit dysfunction was reported during follow-up. Kaplan–Meier analysis demonstrated that the tricuspid conduit group maintained 100% freedom from dysfunction during the 24-month follow-up period. In contrast, the bicuspid group had rates of 90%, 87%, and 83% at 6, 12, and 24 months, respectively (log-rank *p* = 0.016). **Conclusions:** The ePTFE valved conduits provide significant advantages in terms of durability, biocompatibility, cost-effectiveness, and hemodynamic performance for right ventricular outflow tract reconstruction in pediatric cardiac surgery. The findings of our study suggest that tricuspid valve design offers better potential for preventing conduit dysfunction.

## 1. Introduction

Some congenital heart diseases necessitate the use of valved conduits to maintain continuity between the right ventricular outflow tract (RVOT) and pulmonary artery. This subgroup of patients includes those with pulmonary atresia, truncus arteriosus, ventriculoarterial discordance, and variants of tetralogy of Fallot, where the natural course between the right ventricle and the pulmonary artery is absent or inadequate. One of the most important goals of surgical treatment in this subgroup is to create a pathway from the right ventricle to the pulmonary artery that does not cause stenosis during cardiac systole and includes a competent valve that prevents blood from escaping into the right ventricle during diastole. Over the years, various materials and designs have been developed to improve durability, biocompatibility, and hemodynamic performance [1]. Although homografts and xenografts are extensively used, their lifespan is limited, particularly owing to immune-mediated degeneration and calcification [2,3].

Expanded polytetrafluoroethylene (ePTFE) has emerged as a promising material, offering resistance to cellular penetration and subsequent degeneration and calcification due to its low antigenicity, excellent biocompatibility, and microporous structure [4]. Since Yamagishi first described RVOT reconstruction in 1993, using mono-or bicuspid ePTFE valves sutured onto a transannular patch, various designs have been introduced to optimize the hemodynamic performance and longevity of these conduits [5].

This study aimed to compare bicuspid and tricuspid ePTFE valve conduits in terms of structural design, hemodynamic performance, and clinical outcomes. By analyzing the available data from the existing literature and clinical experience, we sought to clarify the relative advantages and limitations of each design. To the best of our knowledge, this is the first study to compare the outcomes of bicuspid and trisucpid handmade ePTFE conduits.

## 2. Materials and Methods

The medical records of 72 consecutive patients who underwent implantation of a handmade bicuspid or tricuspid valve ePTFE conduit between March 2021 and May 2024 were reviewed retrospectively. Data were collected retrospectively from patients’ previous hospital records. This study was approved by the institutional ethics committee on 29 November 2022 (E-28001928-604.01.01-332) and was conducted according to the principles of the Declaration of Helsinki.

This study included all patients treated with handmade valve conduits. 0.1 mm PTFE membrane was used for the leaflets, which were mounted into PTFE tubes to form conduits. The methods described by Yoshida et al. were followed for bicuspid conduits, whereas tricuspid conduits were constructed using our own templates with the same material [6]. The patients who underwent conduit replacement with bovine jugular vein (BJV), bovine or porcine pericardial conduits with PTFE membrane leaflets, or ePTFE conduit in pericardial membrane leaflets and those in the neonatal age group were excluded from the study. In addition, fewer than a dozen cases were excluded from the study, as they were created using a different template by a surgeon who is currently not affiliated with our center. Patients included in the study were divided into two groups according to their valve configurations.

### 2.1. Surgical Technique

**Figure 1:**
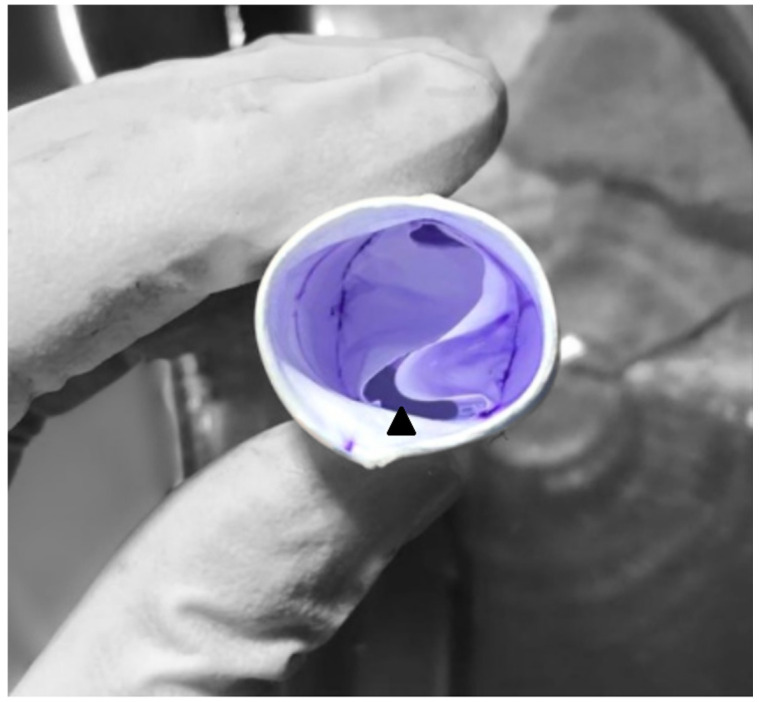
Handmade ePTFE bicuspid valved conduit. The posterior part of the conduit contains a small non-valved portion (about 15% of the circumference), indicated with an arrowhead.

**Figure 2:**
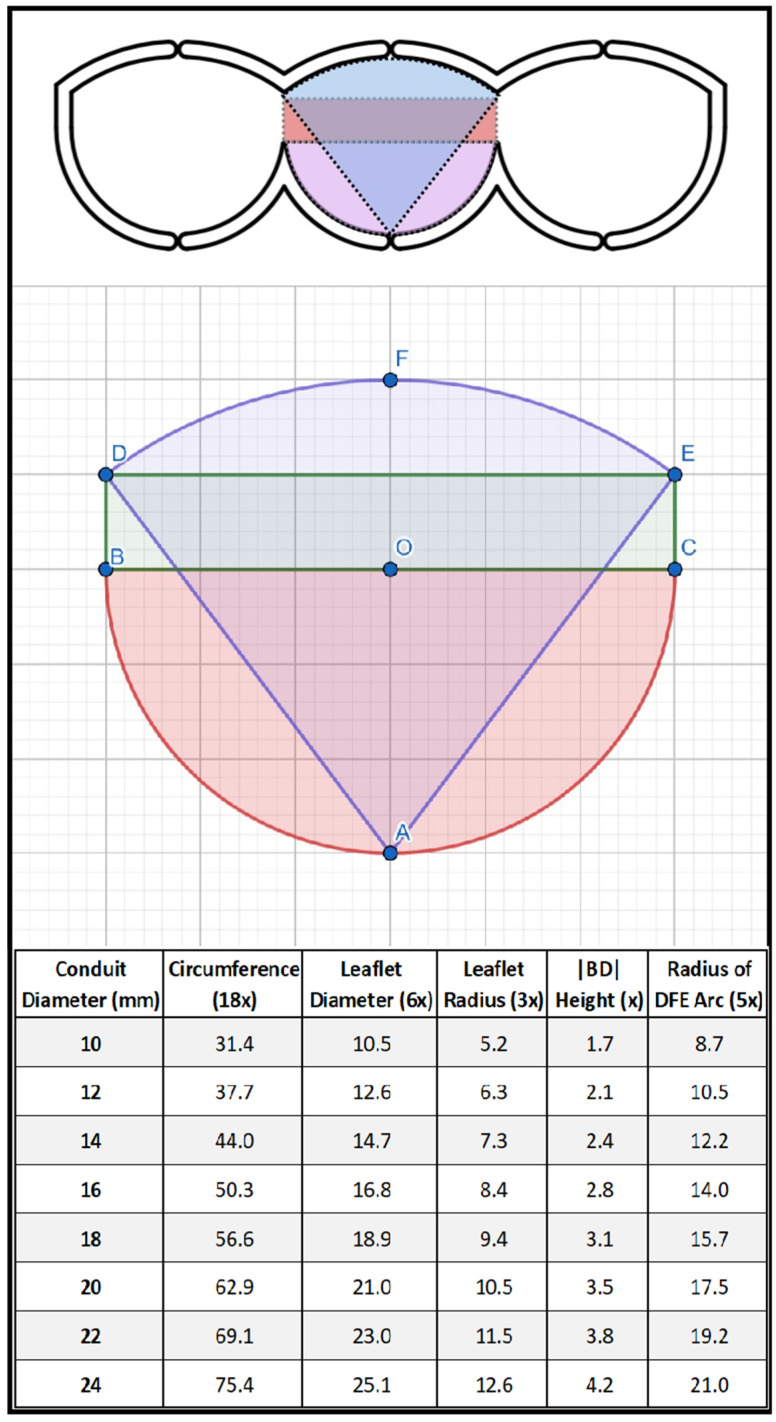
The geometric background and schematic view of our tricuspid template. The exact internal dimensions of the template have been designed as follows. A single leaflet is composed of three separate geometric shapes: a semicircle (with the center “O”) with a diameter of six units at the base; a rectangular section (B, C, E, D) with a height of one unit that provides the commissural height; and a fan connecting points “D and E” that reaches a height of one unit at its highest point for ensuring stability and secure coaptation at the center of the conduit when the valves are in the closed position. The slope of the fan’s (D, F, E) arc was determined based on the projection of a circle segment with a diameter of five units, originating from point “A”. When designing the template, we placed the three leaflets side by side and created a 1.5 mm thick groove around the calculated exact dimensions on the plate to mark the cutting line.

**Figure 3:**
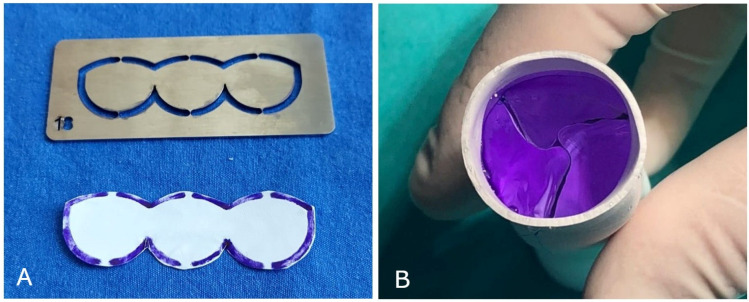
(**A**) The mold and trimmed tricuspid valve from 0.1 mm thick PTFE membrane. When cutting the PTFE membrane, a 1.5 mm marked line is also trimmed from the outer margin, which is used as the stitching line of the leaflets. In this way, a possible reduction in the functional size of the leaflets is prevented. (**B**) The final view of the valved conduit.

The ePTFE valved conduit was prepared on a sterile back table during anesthesia induction in the operating room. While creating a bicuspid conduit, we used the method described by Yoshida et al. [6]. The cusps were shaped from a 0.1 mm thick PTFE membrane according to the dimensions (width, height, and fan size) outlined in a previous study [6]. Initially, the ePTFE tube was inverted inside-out, and the cusps were sutured with 6.0 polypropylene to the inside wall of the ePTFE conduit. In this technique, the cusps are positioned to form only the anterior commissure, and a narrow nonvalved portion is left posteriorly (Figure 1). After the conduit was reverted to its original inside-out configuration, horizontal commissuroplasty sutures, also referred to as memory sutures, were placed to reinforce the commissures and to ensure optimal coaptation, and the usual saline test was applied as a safety measure to confirm valve function. To create a tricuspid conduit with the same material, custom templates were employed to shape 0.1 mm ePTFE leaflets, which were then sutured inside the ePTFE tube to create a conduit with three semilunar cusps, using the same technique as for the bicuspid conduits (Figure 2). Commissures were reinforced and resuspended using horizontal commissuroplasty sutures to enhance strength and ensure optimal coaptation. Valve coaptation was confirmed using the saline test (Figure 3).

### 2.2. Echocardiographic Evaluation

All the patients underwent transthoracic echocardiography before discharge and during the follow-up period. The grade of PTFE conduit stenosis was evaluated by measuring the peak velocity through the valve with continuous wave Doppler and classified as mild if the peak gradient was less than 36 mmHg, moderate between 36 and 64 mmHg, and severe if it was more than 64 mmHg. The degree of conduit regurgitation was classified as none, trivial (grade 0), mild (grade 1), moderate (grade 2), or severe (grade 3). Conduit dysfunction was defined as moderate or greater regurgitation, or severe stenosis.

### 2.3. Postoperative Antiplatelet/Anticoagulant Therapy

All patients received initial anticoagulant therapy with warfarin (target INR: 1.5–2.5) and acetylsalicylic acid (100 mg) for 3 months, followed by long-term acetylsalicylic acid therapy.

### 2.4. Statistical Analysis

Data analysis was conducted using SPSS version 25.0 software. The normal distribution of variables was examined using histogram graphs and the Kolmogorov–Smirnov test. Descriptive analyses are presented using mean, standard deviation, median, and interquartile range (IQR) values. Comparisons in 2 × 2 tables were performed using Pearson’s chi-square test. For non-normally distributed (non-parametric) variables, the Mann–Whitney U test was used to evaluate differences between groups. Kaplan–Meier analysis was performed to evaluate the follow-up results of bicuspid and tricuspid conduits, and the groups were compared using the log-rank test. Conduit dysfunction was defined as moderate or severe pulmonary regurgitation during follow-up. The time-to-event period was defined as the period from the date the conduit was first inserted to the date when conduit dysfunction was first detected echocardiographically, the conduit was removed for another reason, or the patient died. Cases where the *p*-value was below 0.05 were considered statistically significant.

## 3. Results

A total of 72 patients underwent cardiac surgery involving handmade PTFE valved conduit replacement. A bicuspid handmade valve conduit was used in 30 patients (41.7%), and a tricuspid handmade valve conduit was used in 42 patients (58.3%). The female-to-male ratio was 30/42. The median age of the patients was 69 months (IQR: 26–123), and the mean weight of the patients was 21.5 ± 15.1 kg. The most common diagnosis was pulmonary atresia (PA) with ventricular septal defect (VSD) in 36 patients (50%). Thirty-seven (51.4%) patients underwent conduit exchange surgery. In the preoperative echocardiography of patients who underwent conduit exchange, the mean peak gradient was 70 ± 30 mmHg, measured from the failed conduit. Thirty-two patients (44.4%) required extensive pulmonary artery reconstruction as part of the surgical procedure. In 35 patients (48.6%), right ventricle-pulmonary artery (RV-PA) continuity was achieved for the first time with a valved conduit. In this group, the main surgical procedures were Rastelli repair in 26 (36.1%) patients, Ross–Konno repair in two (2.8%) patients, and Tetralogy of Fallot (TOF) repair with pulmonary valved conduit in four (5.6%) patients. PTFE valved conduits with diameters of 14–22 mm were used in our series. The 16 mm diameter conduit was the preferred size and was used in 31 (43.1%) patients. The mean cardiopulmonary bypass (CPB) and aortic cross-clamp (CC) times were 213 ± 67 min and 118 ± 53 min, respectively. Patient characteristics are listed in Table 1.

In the initial postoperative echocardiography, 18 (25%) patients had mild and 3 (5.6%) had moderate pulmonary regurgitation (PR). In the bicuspid group, 11 (36.7%) patients had mild and 3 (10%) had moderate regurgitation, and 7 (16.7%) patients had mild regurgitation in the tricuspid group, and the difference was statistically significant between the groups (*p* = 0.004). The median peak gradient was 0 (IQR 0–15) mmHg. None of the patients had moderate or severe pulmonary stenosis (PS) at the conduit level. Mild PS was observed in 10 (33.3%) and 15 (35.7%) patients in the bicuspid and tricuspid groups, respectively (*p* = 0.834). The mean right ventricular systolic pressure was 37 ± 14 mmHg. Detailed initial echocardiographic outcomes are presented in Table 2.

In terms of early postoperative complications, 13 (18.1%) patients underwent reexploration for bleeding. Major aortopulmonary collateral artery (MAPCA) closure was performed in one (1.4%) patient due to pulmonary overflow causing early hemodynamic instability. No patient required intervention in the early postoperative period because of conduit dysfunction. Arrhythmia was observed in six (8.3%) patients, two (2.8%) patients required a temporary pacemaker due to a complete atrioventricular block, and four patients (5.3%) had junctional ectopic tachycardia. Postoperative fever was observed in nine (12.5%) patients, and in all patients, the fever was related to infection, which was confirmed by the microbiological culture results. Non-infectious fever was not observed in any patient.

Eight (11.1%) patients required extracorporeal membrane oxygenation (ECMO) support. Two (2.8%) of these patients required ECMO due to low cardiac output syndrome, three (4.2%) due to low oxygen saturation, and three (4.2%) required mediastinal packing to prevent excessive bleeding, and ECMO was applied to obtain hemodynamic stability. Seven of eight (87.5%) patients were weaned from ECMO, and five (62.5%)of them were discharged from the hospital. The median intensive care unit and hospital stay were 6 (IQR: 4–11.5) and 14 (IQR: 9–20) days, respectively. In-hospital mortality was observed in four patients (5.6%). Mortality was not conduit-related in any patient. The postoperative complications are shown in Table 3.

**Figure 4:**
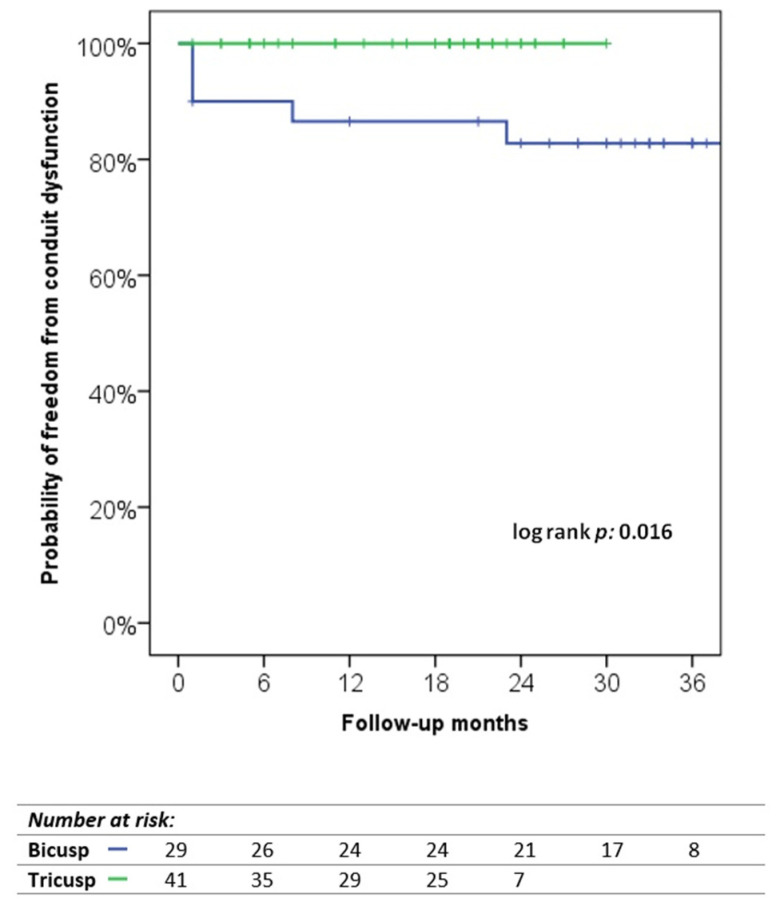
Kaplan–Meier curve for the probability of freedom from conduit dysfunction.

The mean follow-up duration was 22.4 ± 11 months. Four patients (5.9%) underwent balloon angioplasty for peripheral pulmonary branch stenoses. Two patients (2.9%) underwent reoperation for conduit exchange due to late-onset (post-discharge) mediastinitis. One (1.5%) case of late mortality was observed in this cohort. Eleven (40.7%) patients had mild PR, two (7.4%) had moderate PR, and one (3.7%) had severe PR in the bicuspid group, while 10 (24.3%) patients had mild PR in the tricuspid group (*p* = 0.049). The median peak gradient in the valved conduit was 0 (IQR: 0–15) in the bicuspid group and 15 (0–25) mmHg in the tricuspid group, and the difference was statistically significant (*p* = 0.032). The rates of freedom from conduit dysfunction of the ePTFE conduits with bicuspid and tricuspid valves were compared using the Kaplan–Meier analysis(Figure 4). The tricuspid conduit group demonstrated a 100% freedom from conduit dysfunction rate through 24 months of follow-up, which was significantly superior to the 90%, 87%, and 83% freedom from conduit dysfunction rates at 6, 12, and 24 months, respectively (log-rank *p* = 0.016). The final echocardiographic findings are presented in Table 4.

## 4. Discussion

A variety of valved conduits have historically been used for RVOT reconstruction, and several factors influence conduit selection, including durability, size, availability, and cost. Among these, pulmonary homografts, bovine jugular vein grafts, some types of xenografts, and ePTFE valve conduits are currently available [1]. A review of the literature revealed that while there are existing studies on bicuspid and tricuspid valve designs independently, there is a lack of comparative research focusing on the outcomes of the two different techniques [7,8,9,10]. In this study, the use of bicuspid and tricuspid ePTFE valved conduits in pediatric cardiac surgery and the outcomes achieved in our clinic were evaluated. We aimed to summarize and provide insights by integrating the accumulated knowledge with our clinical experience. To the best of our knowledge, our study is the first to compare the outcomes of bicuspid and trisucpid handmade ePTFE conduits.

Pulmonary homografts have traditionally been regarded as the gold standard for RVOT reconstruction in congenital heart surgery and continue to be among the most utilized products [1,11,12,13,14]. Although homografts offer significant advantages, such as their natural biological structure and excellent hemodynamic compatibility, their limitations in durability and restricted availability remain key challenges, particularly in pediatric patients. The cause of conduit dysfunction has been linked to factors such as somatic growth, use of small-sized allografts, and immunologic responses [15,16,17,18]. Mercer et al. compared ePTFE valved conduits and pulmonary homografts and found that they had similar explantation-free rates at five years [19]. Both Hisayuki et al. and Chang et al. reported favorable long-term outcomes with ePTFE valved conduits, highlighting high rates of freedom from reintervention and explantation over extended follow-up periods [20,21]. In the 72 patients included in our study, freedom from explantation due to conduit dysfunction was 100% over an average follow-up period of 22 months, and catheter interventions required in four patients (5.6%) were due to pulmonary artery branch stenosis. The durability of the ePTFE conduit can be attributed to the inert structure of the ePTFE material and its high biocompatibility, which protects it from the inflammatory processes associated with immunological responses [4,21,22].

Xenografts are another widely used alternative conduit replacement. However, they are adversely affected by immune-mediated inflammatory responses triggered by their foreign origin [23,24,25]. In a previous article published by our clinic, we reported unfavorable outcomes associated with the use of a specific brand of xenograft, which was the only available option at that time frame. According to the findings in the study, the use of those “mentioned” xenografts were discontinued due to its non-biocompatible like structure and aggressive immune-reactive response, and valved ePTFE conduits were subsequently introduced into our practice [26]. Notably, none of the patients who received valved ePTFE conduits experienced non-infectious fever, which has been observed in those treated with xenografts. The chemically inert and biocompatible properties of ePTFE contribute to the prevention of immune-mediated inflammatory responses and safeguard the conduit from such reactions [4,21,22].

Bovine jugular vein grafts offer easy access, availability, and small diameter options. However, grafts lacking external support are less resistant to high pressure and are at increased risk of dilatation, especially in patients with pulmonary branch stenosis and pulmonary hypertension [27,28,29]. In a study conducted by Hirai et al., 44 cases using BJV or ePTFE valved conduits were evaluated, and no significant difference was found between the two groups in terms of pulmonary stenosis and pulmonary regurgitation [30]. However, aneurysmal dilatation was observed in 25% of the patients in the BJV group but not in the ePTFE group. Aneurysmal dilatation in BJV conduits is associated with pulmonary branch stenosis [30]. Similarly, in our study, no aneurysmal dilatation was observed in any of the valved ePTFE conduits, including in the four patients who were catheterized due to pulmonary branch stenosis. Currently, as patients with congenital heart disease reach adulthood, clinicians are increasingly encountering individuals with elevated pulmonary artery pressure, including during pregnancy [7]. In this context, it is estimated that the use of an ePTFE conduit reduces the risk of conduit dysfunction during pregnancy. It has also been reported that bovine jugular vein grafts have a high incidence of early and late endocarditis (5% to 10%) [31,32,33,34]. In contrast, ePTFE’s microporous, chemically stable, and biocompatible structure of ePTFE makes it unlikely for microorganisms to adhere to the graft wall or damage its structure through microbiological or enzymatic reactions [35]. In a study by Miyazaki et al. including 902 patients, the conduit infection rate requiring ePTFE valve conduit replacement was 0.3% [7]. In our series, two (2.8%) patients underwent surgical intervention due to mediastinitis, during which their conduits were replaced concomitantly. Intraoperative evaluation of these patients revealed no signs of vegetation or endocarditis within the graft lumen and the valves appeared mobile and functional.

In our clinic, ePTFE valved conduits were initially constructed using only the bicuspid technique described by Yoshida et al., which we used as a model in our practice. In our cohort, 30 patients had bicuspid valved conduits with a mean conduit diameter of 17.3 mm. The patient population showed more regurgitant conduits in the bicuspid group. There were five patients with at least moderate valve insufficiency (16.7%) during follow-up but who did not observe moderate or greater valve stenosis in the early and late follow-up. The diameters of the conduits that developed insufficiency consisted of one 14 mm, two 16 mm, one 18 mm, and one 22 mm graft, so clearly the insufficiency could not be associated with the graft diameter. Our results were consistent with the series of Yoshida et al.; in their study, 16 mm and larger conduits were used in 18 patients, while the dimensions of the three tube grafts that developed valve insufficiency were not specified [6]. Choi et al. also reported patients with larger-sized conduits which were showing fewer regurgitant valves compared to our series, without any difference in conduit dimensions. We think that the valvular insufficiency of the two patients who had 16 mm tube grafts and the time of insufficiency in the early postoperative period may have been due to technical problems since the grafts were made in the early periods of the study [36]. Quintessenza et al. reported that bileaflet ePTFE valves were applied to 126 patients in the RVOT, and only 4 patients developed moderate or greater pulmonary insufficiency without pulmonary stenosis [37]. In their series comparing bileaflet-valved ePTFE conduits with homografts in patients under 2 years of age, Mercer et al. reported that small-sized ePTFE valved conduits may be an alternative to homografts [19]. Seease et al. similarly reported that bicuspid ePTFE conduits used in neonates demonstrated durability similar to that of homografts [38]. Matsushima et al. reported in their series of 50 patients that bileaflet ePTFE valved conduits with dimensions between 10 and 16 mm can serve as a reliable bridge until the next conduit replacement [39]. Ootaki et al. changed the design of ePTFE valved conduit grafts from bicuspid to tricuspid because they believed that excessive bicuspid leaflet height could impair valve movement [40]. In vitro studies on ePTFE conduits with bileaflet valves are limited, and the model developed by Dur et al., which we used as the bileaflet valve design, was tested [41]. The diastolic regurgitant flow fraction was reported to be 25% in this study, which is consistent with our study on early and mid-term PR formation. The reason for PR was related to the non-valved portion of the conduit, which was 15% of the conduit circumference. In another in vitro study, Matsushima et al. tested bicuspid valve conduits and noted that the peak gradient increased with increasing cardiac output [39].

Subsequently, we incorporated the tricuspid technique with our own template into our practice, suggesting that tricuspid valves might offer greater physiological compatibility, especially in larger grafts. There is a large body of literature on ePTFE conduits with tricuspid valves, and satisfactory results have been reported with long-term follow-ups [42,43]. The ePTFE trileaflet-valved conduit offers several benefits. The symmetrical trileaflet design closely mimicked the natural structure of the pulmonary semilunar valve, significantly reducing the risk of pulmonary valve regurgitation. In a study conducted by Miyazaki et al., it was stated that trileaflet valve conduits with bulging sinuses make the valve function more physiological by reducing the valve closure time and required energy during diastole [44,45]. Although tricuspid ePTFE conduits show a high rate of freedom from PS and PR, multicenter studies have reported that relative conduit stenosis due to somatic growth is the most common reason for the explantation of tricuspid valve conduits [7,20]. In our study, in the early follow-up, no moderate or above PR or PS was observed in any of the tricuspid valve conduits. In the mid-term follow-up, moderate PS was detected in only two (4.8%) patients, which is consistent with the literature.

There are specific intraoperative considerations for the success of ePTFE-valved conduits. Determination of the appropriate size of the valved conduit remains a controversial issue. Patients with a smaller conduit are likely to require earlier surgical intervention owing to somatic growth. However, it has been reported that oversized conduits do not enhance the long-term durability, but in the literature, there is a prevailing preference for larger grafts; however, it should be kept in mind that large grafts can be compressed under the sternum in a non-orthotopic position [46,47]. It has also been shown that large grafts can trigger neointimal proliferation by causing turbulent flow and compression in the distal anastomosis region in patients with small pulmonary branches [48]. We believe that flow dynamics are very important for the success of the graft and the maintenance of valve functions. Hemodynamic studies have shown that flow rate decreases in the small curvature (posterior surface) of the graft [6,41]. Therefore, when placing the conduit, we focused on the posterior placement of one commissure. We kept the valves distally located approximately 1 cm below the anastomosis area and took care to protect them from bending and sternum pressure caused by the angulation of the graft.

One of the most important advantages of using ePTFE valved conduits is their cost effectiveness; as previously reported in the literature, the cost of handmade PTFE valved conduits is less than the existing commercially available valved conduits [21,49]. As well as being more affordable than all other materials, they can be produced in a wide range of sizes, allowing for easy access to products of the desired size.

As a result, the ePTFE conduits with tricuspid valves in our series provided 100% freedom from conduit dysfunction during the 24-month follow-up, indicating the potential advantages of this design in terms of durability and hemodynamic performance. In our opinion, tricuspid valve design may contribute to the prevention of conduit dysfunction as it more closely resembles the natural structure of semilunar valves and supports symmetrical valve motion. However, since our experience with bicuspid valve conduits dates back to earlier periods, it should be noted that early valve insufficiencies observed in this group may be due to technical reasons during the learning curve phase. In the bicuspid group, early conduit insufficiency was observed in three patients, likely due to technical factors. Additionally, two patients who initially showed no insufficiency developed valve insufficiency at 8 and 23 months, respectively. Given that no early- or late-onset significant insufficiencies were observed in the tricuspid group, we propose that the design differences between bicuspid and tricuspid valve conduits may influence the risk of conduit dysfunction.

However, this study has some limitations. Further studies with a larger cohort and long-term follow-up are required to validate these findings. The absence of small-diameter conduits in our study limited our ability to evaluate the effect of the valve design on these small-size grafts. Additionally, the long-term outcomes of the conduit designs used in this study should be further supported by investigations involving larger patient populations.

In conclusion, handmade ePTFE valved conduits offer significant advantages in terms of durability, biocompatibility, hemodynamic performance, and cost-effectiveness for RVOT reconstruction in pediatric cardiac surgery. Our study findings suggest that tricuspid valve design, particularly its closer resemblance to semilunar valve anatomy, may help prevent conduit dysfunction. However, early failures of bicuspid valve design could be attributed to technical factors, which can be minimized with surgical experience. Long-term follow-up and larger studies may further validate ePTFE valve conduits as a reliable and effective treatment option. In addition, the low cost and easy availability of these conduits may make them a more attractive choice for future applications.

## Figures and Tables

**Table 1:** Patients’ characteristics.

	All Patients (*n* = 72)	Bicuspid (*n* = 30)	Tricuspid (*n* = 42)	
Variables	*n* (%)/mean ± SD/med (IQR)	*n*/mean ± SD/med (IQR)/%	*n*/mean ± SD/med (IQR)/%	*p*
Gender				
Female	30 (42%)	13 (43.3%)	17 (40.5%)	0.808
Male	42 (58%)	17 (56.7%)	25 (59.5%)	
Age (months)	69 (26–123)	43 (13–80)	87 (46–149)	0.001 *
Body weight (kg)	21.5 ± 15.1	13.9 ± 6.2	27.1 ± 17.7	<0.001 *
Diagnosis of the Patients’				
Truncus arteriosus	7 (9.7%)	3 (10%)	4 (9.5%)	0.863
Pulmonary atresia with VSD	36 (50%)	15 (50%)	21 (50%)	
TGA with VSD and PS	6 (8.3)	2 (6.7%)	4 (9.5%)	
Tetralogy of Fallot	12 (16.7)	5 (16.7%)	7 (16.7%)	
Tetralogy of Fallot with APV	3 (4.2%)	1 (3.3%)	2 (4.8%)	
LVOTO	4 (5.6%)	1 (3.3%)	3 (7.1%)	
Others	4 (5.6%)	3 (10%)	1 (2.4%)	
CPB time (min)	213 ± 67	191 ± 56	228 ± 70	0.02 *
CC time (min)	118 ± 53	102 ± 51	131 ± 53	0.08
Diameter of PTFE Conduits	16 (16–19)	16 (16–18)	18 (16–20)	0.264
14 mm	7 (9.7%)	5 (16.7%)	2 (4.8%)	
16 mm	31 (43%)	13 (43.3%)	18 (42.9%)	
18 mm	16 (22.2%)	5 (16.7%)	11 (26.2%)	
20 mm	12 (27.8%)	4 (13.3%)	8 (19%)	
22 mm	6 (8.3%)	3 (10%)	3 (7.1%)	

APV: absent pulmonary valve; CC: cross clamp; CPB: cardiopulmonary bypass; IQR: interquartile range; LVOTO: left ventricular outflow tract obstruction; PS: pulmonary stenosis, PTFE: polytetrafluoroethylene; SD: standard deviation; TGA: transposition of great arteries; VSD: ventricular septal defect; * statistically significant.

**Table 2:** Initial postoperative ECHO findings.

	All Patients (*n* = 72)	Bicuspid (*n* = 30)	Tricuspid (*n* = 42)	
Variables	*n* (%)/mean ± SD/med (IQR)	*n*/mean ± SD/med (IQR)/%	*n*/mean ± SD/med (IQR)/%	*p*
Conduit Insufficiency				
Grade 1	18 (25%)	11 (36.7%)	7 (16.7%)	0.004 *
≥Grade 2	3 (4.2%)	3 (10%)	0	
Conduit Stenozis				
Mild	25 (34.7%)	10 (33.3%)	15 (35.7%)	0.834
Moderate	0	0	0	
Severe	0	0	0	
Peak Gradient on Conduit (mmHg)	0 (0–15)	0 (0–15)	0 (0–20)	0, 467
RV systolic pressure (mmHg)	40 ± 16	39 ± 17	41 ± 14	0.215

IQR: interquartile range; RV: right ventricle; SD: standard deviation; * statistically significant.

**Table 3:** Early postoperative complications.

	All Patients (*n* = 72)	Bicuspid (*n* = 30)	Tricuspid (*n* = 42)	
Variables	*n* (%)/mean ± SD/med (IQR)	*n*/mean ± SD/med (IQR)/%	*n*/mean ± SD/med (IQR)/%	*p*
Open Sternum	22 (30.6%)	6 (20%)	16 (38.1%)	0.100
Re-exploration for Bleeding	13 (18.1%)	5 (16.7%)	8 (19.1%)	0.796
Pnömonia	8 (11.1%)	4 (13.3%)	4 (9.5%)	0.612
Mediastinitis	2 (2.8%)	1 (3.3%)	1 (2.4%)	0.808
Septicemia	4 (5.6%)	1 (3.3%)	3 (7.1%)	0.487
Neurologic Event	5 (6.9%)	1 (3.3%)	4 (9.5%)	0.308
Arrhythmia	6 (8.3%)	3 (10%)	3 (7.1%)	0.665
ECMO	8 (11.1%)	4 (13.3%)	4 (9.5%)	0.612
Duration of ECMO (days)	3.5 (2.5–4.5)	4 (2.5–18.5)	3.5 (2.5–4)	0.557
ICU stay (days)	6 (4–11.5)	6 (4–10)	7 (5–13)	0.183
Hospital stay (days)	14 (9–20)	13 (8–15)	15 (11–27)	0.020 *
In-Hospital Mortality	4 (5.6%)	3 (10%)	1 (2.4%)	0.164

ECMO: extra-corporeal membrane oxygenation; ICU: intensive care unit; IQR: interquartile range; SD: standard deviation; * statistically significant.

**Table 4:** Follow-up and final ECHO findings.

	Surviving Patients (*n* = 68)	Bicuspid (*n* = 27)	Tricuspid (*n* = 41)	
Variables	*n* (%)/mean ± SD/med (IQR)	*n*/mean ± SD/med (IQR)/%	*n*/mean ± SD/med (IQR)/%	*p*
Follow-up duration (months)	22.4 ± 11	29.6 ± 11.5	17.3 ± 7.4	0.001 *
Conduit Insufficiency				
Grade 1	21 (30.9%)	11 (40.7%)	10 (24.4%)	0.049 *
≥Grade 2	3 (4.4%)	3 (11.1%)	0	
Conduit Stenozis				
Mild	37 (54.4%)	14 (51.9%)	23 (56.1%)	0.280
Moderate	2 (2.9%)	0	2 (4.9%)	
Severe	0	0	0	
Peak Gradient on conduit (mmHg)	15 (0–25)	0 (0–15)	15 (0–25)	0.032 *
RV systolic pressure (mmHg)	45 ± 17	40 ± 14	46 ± 15	0.024 *
Baloon angioplasty for peripheral PAS	4 (5.9%)	3 (11.1%)	1 (2.4%)	0.301
Reoperations	2 (2.9%)	0	2 (4.9%)	0.360
Late Mortality	1 (1.5%)	0	1 (2.4%)	0.603

IQR: interquartile range; PAS: pulmonary artery stenosis; RV: right ventricle; SD: standard deviation; * statistically significant.

## Data Availability

The datasets generated and/or analyzed during the current study are available from the corresponding author upon reasonable request.
